# The effect of adhesive tape versus endotracheal tube fastener in critically ill adults: the endotracheal tube securement (ETTS) randomized controlled trial

**DOI:** 10.1186/s13054-019-2440-7

**Published:** 2019-05-07

**Authors:** Janna S. Landsperger, Jesse M. Byram, Bradley D. Lloyd, Todd W. Rice, for the Pragmatic Critical Care Research Group, David R. Janz

**Affiliations:** 1 Department of Medicine, Vanderbilt University, T-1218 Medical Center North, Nashville, TN 37232-2650 USA; 2 Department of Respiratory Therapy, Vanderbilt University, T-1218 Medical Center North Nashville, Nashville, TN 37232-2650 USA; 3 T-1218 Medical Center North, Vanderbilt University, Nashville, TN 37232-2650 USA

**Keywords:** Endotracheal tube, Tube fastener, Facial skin tear, Lip ulcer, Critical care, Intensive care units, Mechanical ventilation, Endotracheal tube dislodgement

## Abstract

**Background:**

The optimal securement method of endotracheal tubes is unknown but should prevent dislodgement while minimizing complications. The use of an endotracheal tube fastener might reduce complications among critically ill adults undergoing endotracheal intubation.

**Methods:**

In this pragmatic, single-center, randomized trial, critically ill adults admitted to the medical intensive care unit (MICU) and expected to require invasive mechanical ventilation for greater than 24 h were randomized to adhesive tape or endotracheal tube fastener at the time of intubation. The primary endpoint was a composite of any of the following: presence of lip ulcer, endotracheal tube dislodgement (defined as moving at least 2 cm), ventilator-associated pneumonia, or facial skin tears anytime between randomization and the earlier of death or 48 h after extubation. Secondary endpoints included duration of mechanical ventilation and ICU and in-hospital mortality.

**Results:**

Of 500 patients randomized over a 12-month period, 162 had a duration of mechanical ventilation less than 24 h and 40 had missing outcome data, leaving 153 evaluable patients randomized to tube fastener and 145 evaluable patients randomized to adhesive tape. Baseline characteristics were similar between the groups. The primary endpoint occurred 13 times in 12 (7.8%) patients in the tube fastener group and 30 times in 25 (17.2%) patients in the adhesive tape group (*p* = 0.014) for an overall incidence of 22.0 versus 52.6 per 1000 ventilator days, respectively (*p* = 0.020). Lip ulcers occurred in 4 (2.6%) versus 11 (7.3%) patients, or an incidence rate of 6.5 versus 19.5 per 1000 patient ventilator days (*p* = 0.053) in the fastener and tape groups, respectively. The endotracheal tube was dislodged 7 times in 6 (3.9%) patients in the tube fastener group and 16 times in 15 (10.3%) patients in the tape group (*p* = 0.03), reflecting incidences of 11.9 and 28.1 per 1000 ventilator days, respectively. Facial skin tears were similar between the groups. Mechanical ventilation duration and ICU and hospital mortality did not differ.

**Conclusion:**

The use of the endotracheal tube fastener to secure the endotracheal tubes reduces the rate of a composite outcome that included lip ulcers, facial skin tears, or endotracheal tube dislodgement compared to adhesive tape.

**Trial registration:**

ClinicalTrials.gov NCT03760510. Retrospectively registered on November 30, 2018

## Background

There are many potential complications during endotracheal tube intubation, including laryngeal trauma, bronchospasm, hypotension, hypoxemia, airway perforation, and vertebral column injury [1]. Following intubation for mechanical ventilation, other potential complications may occur, including the development of lip ulcers or skin tears, endotracheal tube dislodgement or advancement, or endotracheal tube plugging or malfunction. Several techniques are utilized in current clinical practice to secure the endotracheal tube [2], in order to maintain a patent airway and prevent complications. Lip ulcers and facial skin tears are infrequent complications of endotracheal tube securement [2–4], but each is associated with increased financial burden and potentially increased length of stay [2]. As hospital-acquired pressure injuries, treatment of these complications is not reimbursed. Endotracheal tube dislodgement sometimes resulting in unplanned extubation, bronchospasm, or tracheal injury is another more common complication of suboptimal tube securement [5–7]. Additionally, endotracheal intubation and mechanical ventilation are associated with ventilator-associated pneumonia. Proper oral hygiene is essential in decreasing the incidence of ventilator-associated pneumonia [8]. Utilizing an endotracheal tube securement technique that enables providers to perform oral hygiene is imperative. Different endotracheal tube securement techniques may have different effects on these complications.

To evaluate the safety and efficacy of endotracheal tube securement techniques, we performed a pragmatic, randomized controlled trial comparing the effect of adhesive tape versus endotracheal tube fastener on complications including lip ulcers, facial skin tears, endotracheal tube dislodgement, and ventilator-associated pneumonia among critically ill adults requiring intubation and mechanical ventilation for at least 24 h.

## Methods

### Study design

The endotracheal tube securement (ETTS) study was a pragmatic, single-center, open-label, randomized trial comparing the effect of adhesive tape versus endotracheal tube fastener (Hollister® AnchorFast Guard) among critically ill adults requiring intubation and mechanical ventilation for at least 24 h (ClinicalTrials.gov identifier: NCT03760510). Both adhesive tape and endotracheal tube holder were being used in the MICU according to the clinician’s choice prior to initiating the study. The study was approved by the Vanderbilt University Medical Center Institutional Review Board with a waiver of consent.

### Study participants

Patients admitted to the MICU from May 17, 2017, to April 14, 2018, who were deemed by their clinical team to require endotracheal intubation and fulfill the inclusion criteria without meeting the exclusion criteria were enrolled and randomly assigned to adhesive tape versus endotracheal tube fastener. If patients were intubated within 12 h of admission to the MICU, they were eligible for inclusion in the study. Patients had to be enrolled in the study for a minimum of 24 h to be included in the analysis to ensure a reasonable amount of time for complications to occur. Patients were excluded if they (1) were intubated greater than 12 h prior to admission to the MICU, (2) had oral mucosa or facial skin breakdown prior to enrollment, (3) required nasotracheal intubation, (4) had a documented allergy to tape, (5) were pregnant, or (6) were prisoners.

### Randomization

Patients were randomized to endotracheal tube holder or tape in a 1:1 ratio using random permuted computer-generated blocks of 2, 4, and 6. Prior to initiation of the study, randomization assignments were placed in sequentially numbered opaque envelopes, which remained sealed until the decision was made to enroll a patient in the study. Once it had been determined by the treating team that a patient was eligible for the study (i.e., intubated less than 12 h), the operator opened the envelope and followed the assignment of either adhesive tape or endotracheal tube fastener.

### Study treatments

Prior studies investigating endotracheal tube securement techniques require that the endotracheal tube be repositioned and re-taped every 24 h. However, due to the pragmatic design and intent of this protocol, the endotracheal tube was not repositioned as part of the study protocol in either group. In both groups, the tube was repositioned as needed according to ICU policy or protocols or at the discretion of the provider, bedside nurse, or respiratory therapist, per usual practice. For both groups, oral hygiene was performed every 12 h and oral moistening every 2 h based on ICU policy.

### Data collection

Data were collected prospectively at the time of intubation and from the medical record in order to determine the effect of the assigned intervention on short- and long-term outcomes. All data were collected non-invasively and were already a part of clinical data obtained in usual ICU care at the bedside or in the medical record. Due to the pragmatic nature of the trial, no additional data were collected that were not observed at the bedside or obtained from the medical record.

Age, gender, height, weight, race, active medical problems at the time of intubation, active comorbidities complicating intubation, indication for intubation, whether a reintubation, and whether the face was soiled during the intubation were all collected at the time of intubation. Depth of the tube as measured at the lip line and position in the mouth (i.e., 22 cm at the lip, midline) at time of securement was also recorded. The following in-hospital outcomes were recorded via electronic medical record review: days on mechanical ventilation, and vital status at the time of ICU and hospital discharge. Oral mucosa assessment, facial skin integrity assessment, frequency of endotracheal tube repositioning, and endotracheal tube dislodgements—defined as either complete dislodgement of the endotracheal tube (i.e., accidental extubation) or needing to reposition the endotracheal tube at least 2 cm (i.e., need to pull ETT back > 2 cm or move it down > 2 cm)—were all collected in duplicate using bedside sheets completed by nursing and respiratory therapy and electronic medical record review. In addition, as per usual practice in our ICU, facial skin tears and lip ulcers were independently assessed and confirmed by the nursing quality improvement associate, who was not part of either the patient care or the study team. Endotracheal tube dislodgements were retrospectively confirmed by a study personnel via manual chart review.

### Study outcomes

The primary outcome measure was a composite of any of the following: development of lip ulcer, ventilator-associated pneumonia, endotracheal tube dislodgement, or facial skin tears from the time of randomization to the earlier of death or 48 h after extubation. Secondary outcome measures included each of the individual components of the primary composite outcome (i.e., presence of lip ulcer, endotracheal tube dislodgement, ventilator-associated pneumonia, or facial skin tears), duration of mechanical ventilation, and ICU and hospital mortality.

### Statistical analysis

#### Sample size determination

We estimated the expected incidences of tube dislodgement and lip ulcer development to be 20 and 1.1 per 1000 ventilator days, respectively, based on the data from the previous 12 months. Using the sum of these overall incidences (i.e., 21.1 per 1000 ventilator days) and a standard deviation of 15 per 1000 ventilator days, PS software [9] calculated a need for 142 evaluable patients in each arm to detect a clinically meaningful change of 5 episodes per 1000 ventilator days with 80% power at a two-sided alpha level of 0.05. To account for the loss of patients not ventilated for 24 h and a 5.6% dropout or loss to follow-up rate, we planned to enroll 500 patients to achieve the 284 eligible patients needed for the study.

#### Analysis principles

The primary analysis was conducted on an intention-to-treat basis (patients with protocol violations were analyzed per the assigned treatment arm). All hypothesis tests were two-sided, with an *α* of 0.05 unless otherwise specified. The primary endpoint was the continuous variable of incidences of lip ulcers, tube dislodgement, or ventilator-associated pneumonia per 1000 ventilator days. The differences between the two groups were compared using Mann-Whitney *U* test. We conducted an unadjusted analysis examining the treatment effect of endotracheal tube securement method on each of the pre-specified secondary and tertiary outcomes. Continuous outcomes were compared with the Mann-Whitney *U* test and categorical variables with the chi-square or Fisher exact test as appropriate. Due to the differences in age in baseline demographics, a post hoc ordinal regression was undertaken with age and randomization group as independent variables and rate of composite endpoint per 1000 ventilator days as the dependent variable. All analyses were conducted using SPSS version 25 (IBM SPSS Statistics for Windows, Version 25.0. Armonk, NY).

## Results

### Baseline characteristics

**Fig. 1:**
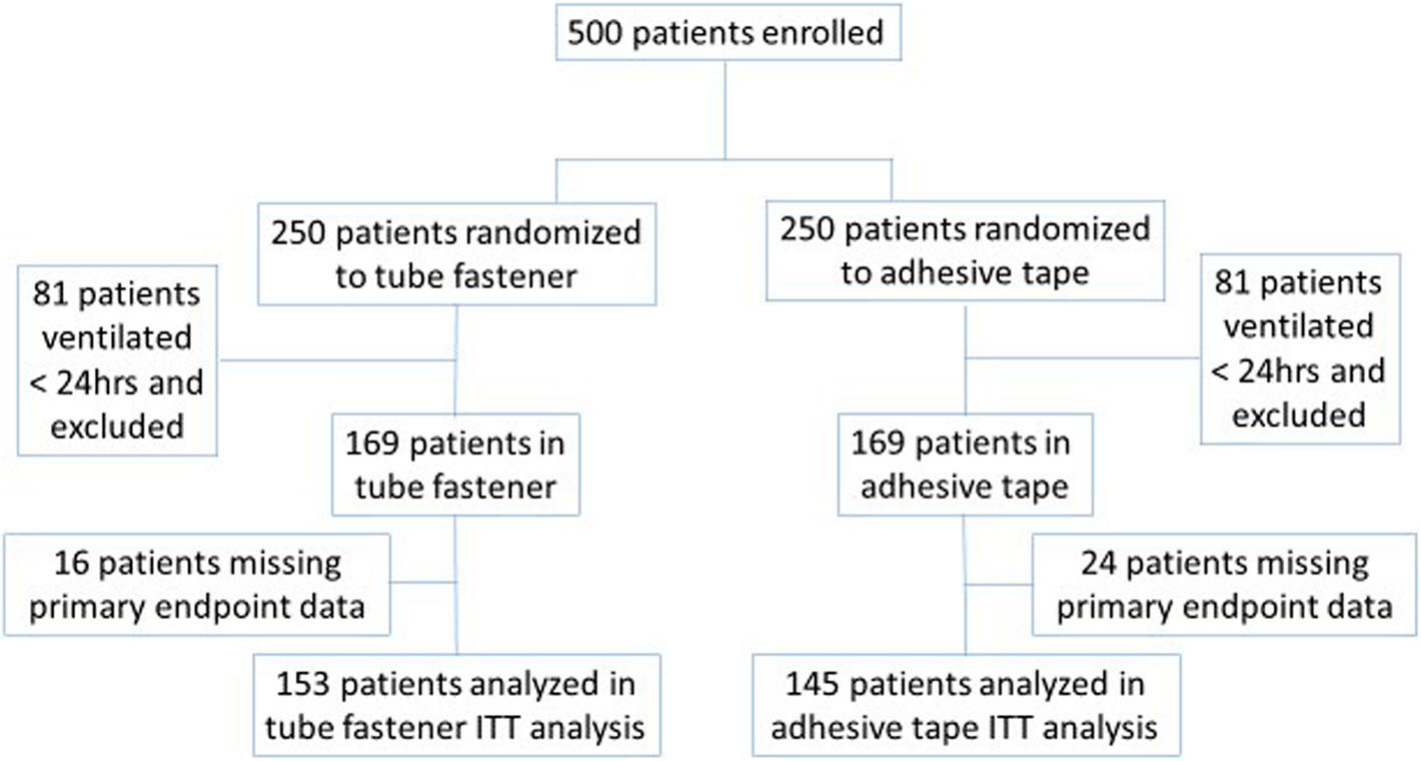
Inclusion and enrollment of patients. CONSORT diagram showing the enrollment of patients into the endotracheal tube securement (ETTS) randomized controlled trial

Of 500 patients randomized, 162 were deemed excluded from the analysis due to the duration of mechanical ventilation less than 24 h and 40 had missing outcome data, leaving 298 in the analysis. One hundred fifty-three were randomized to the tube fastener and 145 were randomized to adhesive tape (Fig. 1). There were no significant differences in baseline characteristics between the patients assigned to receive tube fastener and those assigned to receive adhesive tape (Table 1). The most common indication for intubation among both groups was respiratory failure (45.7% versus 53.1%, respectively). Total ventilation time for all 153 patients in the tube fastener group was 590 days (mean duration 3.9 ± 3.0 days) compared to 570 days for the 145 patients in the tape group (mean duration 3.9 ± 3.4 days) (*p* = 0.75).

**Table 1:** Characteristics of patients at baseline

Characteristic	Tube fastener (*n* = 153)	Adhesive tape (*n* = 145)	*p* value
Age (years)	53.2 ± 16.4	58.5 ± 16.1	0.01
Male sex—no. (%)	77 (50.3)	79 (54.5)	0.47
Race—no. (%)			
Caucasian	122 (79.7)	111 (76.5)	0.89
Black	19 (12.4)	23 (15.8)	
Height (cm)	169.6 ± 11.0	168.7 ± 12.0	0.51
Weight (kg)	86.7 ± 29.6	87.1 ± 31.2	0.91
BMI	30.3 ± 10.7	30.8 ± 11.1	0.69
APACHE II	26.0 ± 8.9	27.4 ± 8.7	0.16
Indication for intubation—no. (%)			
Respiratory failure	70 (45.7)	77 (53.1)	0.52
Altered mental status	36 (23.5)	31 (21.3)	
Airway patency	45 (29.8)	35 (24.1)	
Shock	2 (1.4)	2 (1.4)	
ETT depth at the lip (mean ± std. deviation)	22.7 ± 1.1	22.8 ± 1.2	0.740
Comorbidities complicating intubation			
Upper gastrointestinal bleed—no. (%)	2	1	0.59
Spinal cord injury—no. (%)	0	1	0.30
Vomiting—no. (%)	3	2	0.70

### Primary outcome

**Fig. 2:**
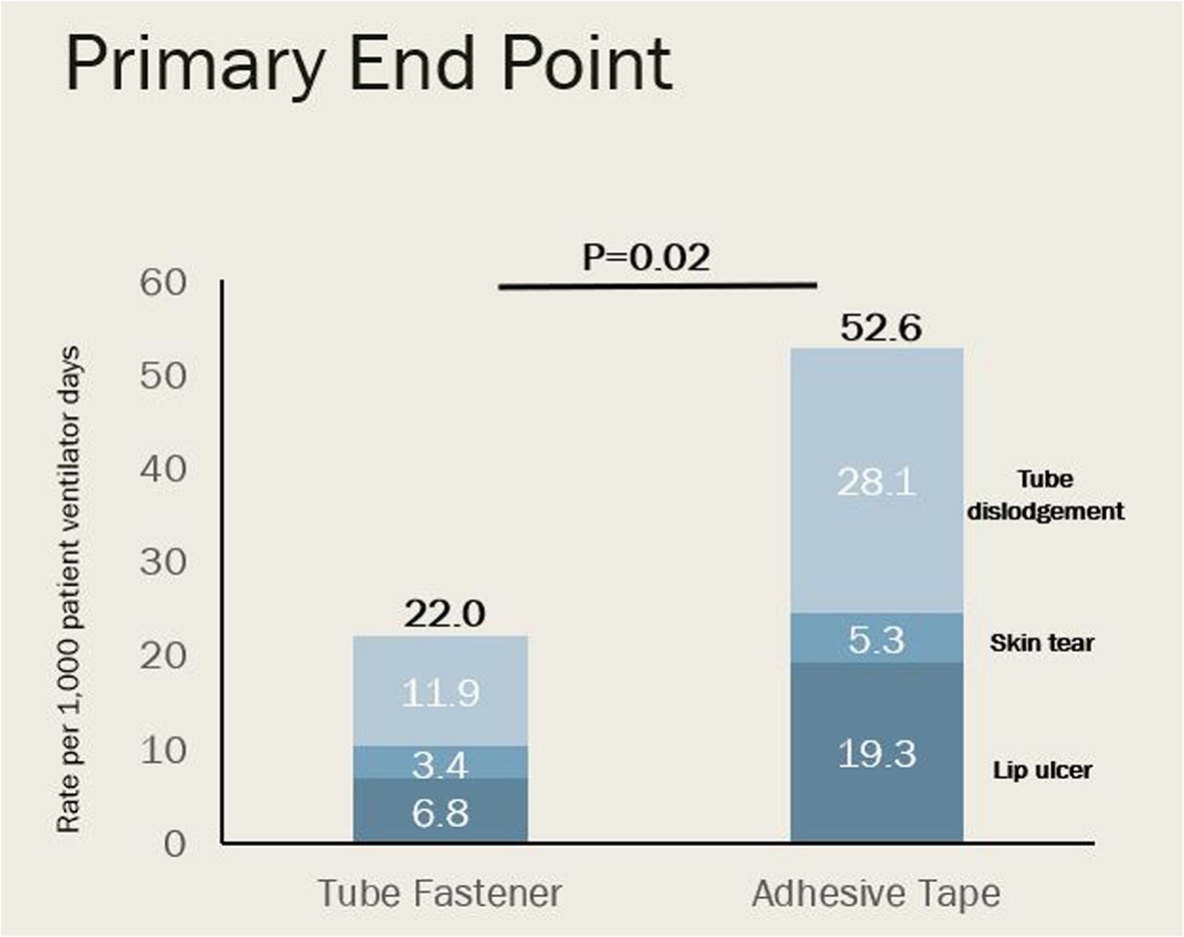
Primary endpoint. The composite of tube dislodgement, lip ulcer, and skin tear per 1000 patient ventilator days was significantly lower in the endotracheal tube fastener group compared to that in the adhesive tape group (*p* = 0.017). There were no incidences of VAP

The primary endpoint, a composite of presence of lip ulcer, endotracheal tube dislodgement, ventilator-associated pneumonia, or facial skin tears from the time of randomization to the earlier of death or 48 h after extubation, occurred 13 times in 12 (7.8%) patients in the tube fastener group and 30 times in 25 (17.2%) patients in the adhesive tape group (*p* = 0.014). The overall rates of the development of the composite endpoint were 22.0 (95% CI 16.3 to 27.7) versus 52.6 (95% CI 47.4 to 57.8) per 1000 patient ventilator days (*p* = 0.020) in the tube fastener and adhesive tape groups, respectively (Table 2; Fig. 2). Univariate analysis demonstrated an OR of 2.33 (95% CI 1.13–4.83; *p* = 0.022) for the development of the composite endpoint when adhesive tape compared to tube fastener was used for endotracheal tube securement. Multivariate analysis including age as a covariate demonstrated no independent association between age and development of the composite endpoint (OR 1.00; 95% CI 0.98–1.02; *p* = 0.84) and similar OR for the development of the composite endpoint in adhesive tape versus tube fastener (OR 2.32; 95% CI 1.11–4.76; *p* = 0.026).

**Table 2:** Clinical outcomes

Outcome	Tube fastener (*n* = 153)	Adhesive tape (*n* = 145)	*p* value
Primary outcome			
Lip ulcers, skin tear, tube dislodgement, or ventilator-associated pneumonia—no. of patients (%)	12 (7.8)	25 (17.2)	0.014
Rate of primary outcome (per 1000 patient ventilator days) (95% CI)	22.0 (16.3–27.7)	52.6 (47.4–57.8)	0.020
Components of primary outcome			
Lip ulcer—no. (%)	4 (2.6)	11 (7.3)	0.050
Rate per 1000 patient ventilator days	6.8 (5.6–8.0)	19.3 (17.1–21.6)	0.052
Skin tear—no. (%)	2 (1.4)	3 (2.1)	0.610
Rate per 1000 patient ventilator days	3.4 (2.0–4.8)	5.3 (4.7–5.9)	0.622
Tube dislodgement*—no. (%)	6 (3.9)	15 (10.3)	0.030
Rate per 1000 patient ventilator days	11.9 (6.5–17.3)	28.1 (24.4–31.8)	0.035
Secondary outcomes			
ETT repositioned—no. (%)	17 (12.1)	40 (29.0)	< 0.001
Self-extubations—no. (%)	2 (1.3)	2 (1.4)	0.957
Ventilator-associated pneumonia	0 (0)	0 (0)	N/A
MV duration (days)	3.9 ± 3.0	3.9 ± 3.4	0.75
ICU mortality—no. (%)	52 (34.0)	51 (35.2)	0.83
Hospital mortality—no. (%)	57 (37.3)	54 (37.2)	0.99

Data are reported as no. (%), rate per 1000 patient ventilator days (95% CI), or mean ± standard deviation

*Tube dislodgement defined as or needing to reposition the endotracheal tube more than 1 cm

### Secondary outcomes

Lip ulcer occurred in 4 (2.6%) versus 11 (7.3%) (*p* = 0.05) patients for rates of 6.8 (95% CI 5.6 to 8.0) versus 19.3 (95% CI 17.1 to 21.6) per 1000 patient ventilator days (*p* = 0.052) while facial skin tears occurred in 2 (1.4%) versus 3 (2.1%) patients for rates of 3.4 (95% CI 2.0 to 4.8) versus 5.3 (95% CI 4.7 to 5.9) per 1000 patient ventilator days (*p* = 0.622) in the fastener and tape groups (*p* = 0.61), respectively. The endotracheal tube was dislodged 7 times in 6 (3.9%) patients in the tube fastener group and 16 times in 15 patients (10.3%) in the adhesive tape group (*p* = 0.030), with rates of 11.9 (95% CI 6.5 to 17.3) versus 28.1 (95% CI 24.4 to 31.8) per 1000 patient ventilator days (*p* = 0.035). One of the tube dislodgements was a self-extubation in 2 patients in each group. There were no occurrences of ventilator-associated pneumonia in either group. ICU mortality was similar, with 52 patients (34.0%) in the tube fastener group and 51 patients (35.2%) in the adhesive tape group dying before ICU discharge (*p* = 0.83). Hospital mortality was also similar between the 2 groups with 57 patients (37.3%) in the tube fastener group and 54 (37.2%) in the adhesive tape group dying prior to hospital discharge (*p* = 0.99).

## Discussion

This study demonstrated that endotracheal tube fasteners reduced the incidence of lip ulcers, skin tears, and tube dislodgement compared to adhesive tape. Although both tube fasteners and adhesive tube have been used to secure endotracheal tubes in patients in clinical practice for several years, few trials have directly compared the effects of these different securement techniques on patient safety and clinical outcomes [2]. While many respiratory therapists may have a personal preference towards one particular securement technique, usually due to ease of use, superiority of one technique over another with regard to outcomes has not previously been demonstrated. Prior studies investigating different endotracheal securement techniques are not generalizable to patients mechanically ventilated in an ICU. In 2007, Carlson et al. examined the force required to extubate endotracheal tubes from cadavers with either tape or endotracheal tube fastener [5]. Results showed that tape required a significantly larger force to extubate the cadavers compared to fastener. However, given this study was undertaken in cadavers, it is unclear if these results translate to adult patients in the ICU. Additionally, in 2014, Mohammed and Hassan demonstrated that securement with twill decreased endotracheal tube slippage in the first 120 min post intubation compared to tape and tube fastener [2]. Failure to evaluate the performance of the securement technique beyond 2 h limits the interpretation of these data into critical care environments where the average duration of mechanical ventilation is measured in days or weeks.

In the current trial, the use of a tube fastener resulted in 7.2% fewer patients developing complications compared to adhesive tape. Overall, using the tube fastener resulted in 19.3 fewer complications per 1000 ventilator days than using adhesive tape. These results suggest that the use of an endotracheal tube fastener rather than adhesive tape will result in fewer lip ulcers, endotracheal tube dislodgements, and facial skin tears. While there was no difference between the groups in clinical outcomes, including duration of mechanical ventilation and ICU or hospital mortality, reducing the rate of lip ulcers, endotracheal tube dislodgements, and facial skin tears will improve patient safety and experience in the ICU and also likely decrease the cost of care. In addition, lip ulcers and skin tears are considered “never events” by payer sources, meaning that treatment of these complications is not reimbursed as part of patient care costs.

The trial has limitations. Conduct at a single academic center limits generalizability. For obvious reasons, the trial was open-label and not blinded, with investigators and clinical personnel aware of the allocation group. However, endpoints were objective and strictly defined, thus limiting the subjectivity of the evaluation. In addition, the composite outcomes of the primary endpoint were independently confirmed by either the quality nurse associate or study personnel. Despite this, it remains possible that some events (skin tears, lip ulcers, ETT dislodgement) may have gone undocumented by the bedside nurse or respiratory therapist and missed by the independent quality nurse associate or study personnel adjudicators. While the securement device may have obscured some lip ulcers or skin tears while it was being used, all patients were assessed for these complications both daily and when the securement device was ultimately removed. Finally, while the overall follow-up was excellent, the pragmatic nature resulted in 40 randomized patients being excluded due to missing primary outcome data. This 0.8% loss to follow-up rate was considerably less than anticipated when calculating the sample size needed to power the study and is unlikely to have significantly altered the results. The composite endpoint would have needed to occur in almost 20% of the 16 patients randomized to tube fastener with missing envelopes, a rate almost three times higher than that occurred in the other patients enrolled in the tube fastener arm of the study.

This trial also has several strengths. It is the first large, pragmatic, randomized trial of different endotracheal tube securement techniques focusing on the complications throughout the duration of mechanical ventilation and clinical outcomes during the ICU stay. The trial was led by an advanced practice provider and run by respiratory therapists. The pragmatic nature of the trial allows for the enrollment of a heterogeneous population of consecutive critically ill patients, and the pragmatic design, without artificially regulating routine endotracheal tube position changes, reflects actual clinical practice, rendering the results more generalizable to the care of critically ill patients in general.

## Conclusion

In conclusion, in this trial involving critically ill adults, securement of an endotracheal tube with a tube fastener resulted in a lower incidence of and fewer patients experiencing lip ulcers, endotracheal tube dislodgements, or facial skin tears compared to securement with adhesive tape.

## Abbreviations

ETT: Endotracheal tube
ETTS: Endotracheal tube securement
ICU: Intensive care unit
MICU: Medical intensive care unit
